# One strain may hide another: Cryptic male-killing *Wolbachia*

**DOI:** 10.1371/journal.pbio.3002076

**Published:** 2023-03-30

**Authors:** Emily A. Hornett, Gregory D. D. Hurst

**Affiliations:** 1 Department of Evolution, Ecology and Behaviour, Institute of Infection, Veterinary and Ecological Sciences, University of Liverpool, Liverpool, United Kingdom; 2 Vector Biology, Liverpool School of Tropical Medicine, Liverpool, United Kingdom

## Abstract

While heritable symbionts are common in insects, strains that act as male-killers are considered rare. This Primer explores a new study in PLOS Biology which reveals a novel male-killer hidden by co-infection and host resistance, highlighting the complexity of host-microbial interactions in natural systems.

## Teaser

While heritable symbionts are common in insects, strains that act as male-killers are considered rare. A new study in *PLOS Biology* identifies a novel male-killer hidden by coinfection and host resistance, highlighting the complexity of host-microbial interactions in natural systems.

Heritable endosymbionts are common in arthropods. For instance, in the Darwin Tree of Life project, sequencing of a single individual from 368 insect species revealed 93 to be infected with at least one strain of the heritable bacterium, *Wolbachia* [1]. This number is likely an underestimation of *Wolbachia* incidence, as sampling a single individual from a species may miss *Wolbachia* infections not present in all individuals. Aside being common, symbiont infection is an important component of host biology for two reasons. First, transmission of the symbiont from host mother to progeny selects for the symbiont to contribute beneficially to host function—a healthy female host is also a transmitting host. Second, the strictly maternal pattern of inheritance—where the symbiont passes into the egg of infected mothers (but not through sperm of infected fathers) makes male hosts “dead ends,” leading to heritable microbes evolving an array of reproductive manipulation phenotypes. These “reproductive parasitisms” are of two types. In the first type, maternally inherited microbes distort the host sex ratio through male-killing (MK), feminisation of genetic males, or parthenogenesis induction—acts that promote the production or survival of female hosts. The second type is particularly common and involves the induction of cytoplasmic incompatibility (CI) phenotypes, where zygotes formed from a mating between infected males and uninfected (or differently infected) females die. Both beneficial and reproductive parasitic impacts drive various features of host ecology and evolution, from dietary breadth through natural enemy resistance, to patterns of sexual selection and diversity in sexual reproduction.

**Fig 1:**
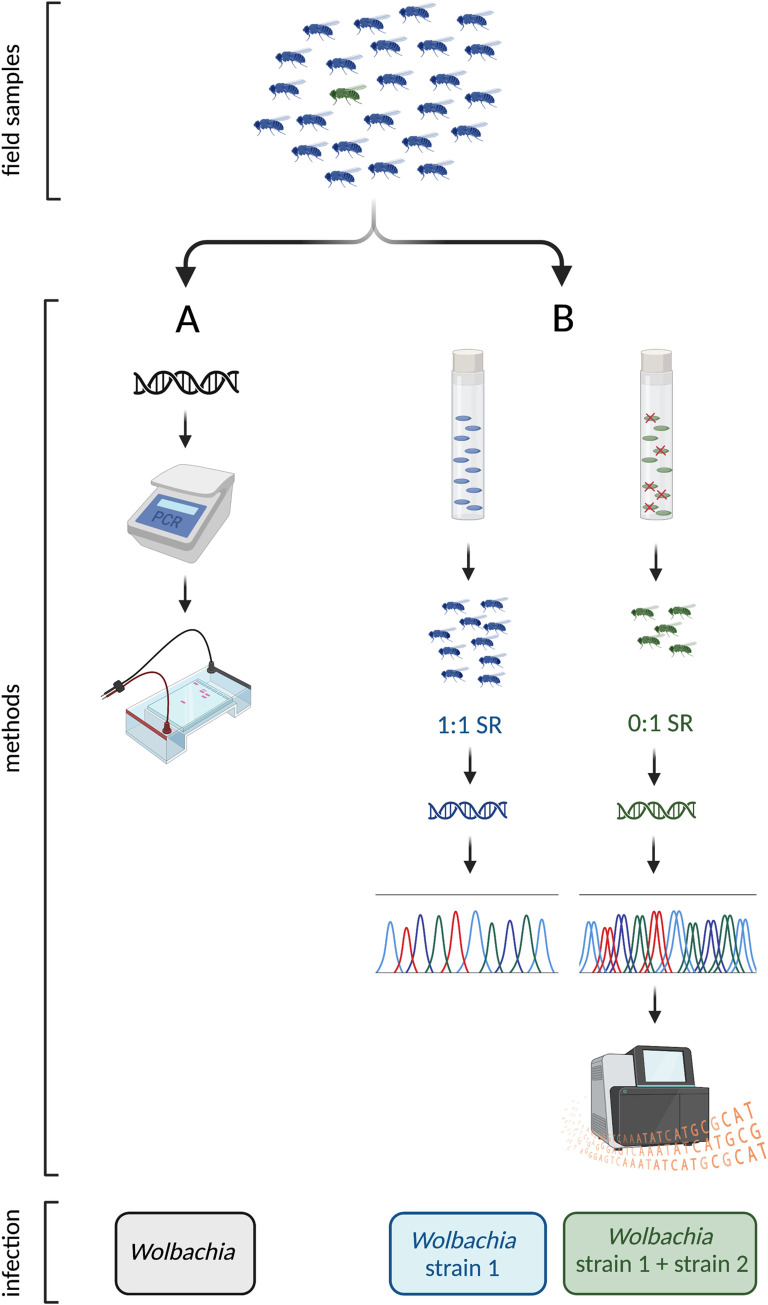
Traditional nonspecific PCR assays for symbiont infection will miss rare coinfections. In this graphic, the natural population comprises flies either with a single infection (blue: strain 1) or a double infection including the male-killer (green: strain 1 + strain 2(MK)). (**A**) In a PCR survey, both male and female individuals test positive for *Wolbachia* because of the high prevalence non-MK *Wolbachia* infection, obscuring evidence of MK in the population. (**B**) In the current paper, female flies from natural populations are allowed to individually oviposit. Egg hatch rate is noted (embryonic MK results in the death of half of the embryos; late MK results in the death of larvae—as observed in the current study). The sex ratio (SR) of the surviving adult progeny is determined (a female bias (0:1) is produced upon infection with a MK). When DNA is extracted from these flies and the *Wolbachia* amplicon Sanger sequenced, double peaks suggest coinfection. Genome sequencing confirms the presence of >1 strain of *Wolbachia*, the second strain being the male-killer. Figure created with BioRender.com.

MK was the last of the *Wolbachia* sex ratio distorting phenotypes to be discovered [2]. While the number of records of MK has grown in recent years, it is still considered to be relatively infrequent. A new study in *PLOS Biology* reports the discovery of a novel male-killer and highlights additional factors explaining why MK incidence may have been underestimated [3]. Richardson and colleagues studied Australian populations of the fruit fly *Drosophila pseudotakahashii*, a species previously known to harbour a CI-inducing *Wolbachia* strain in the majority of individuals [4]. In the current work, they identified an additional low prevalence MK *Wolbachia* coinfecting individuals through breeding 188 female field collected flies. In past research, detection of low prevalence MK infections in species that are not easily lab culturable has relied on PCR assays, with presence of sex ratio distortion inferred by symbiont infection in female but not (or rarely) in male hosts [5]. The new study suggests that binomial PCR assays (presence/absence of a PCR product) will miss infections; in *D*. *pseudotakahashi*, both male and female individuals would return PCR positive for the CI *Wolbachia*, hiding the rare coinfection with the MK strain (Fig 1). In his poem “*One train may hide another*,*”* the poet Kenneth Koch relates how something that is very evident may cause us to overlook alternate versions of the same [6]. In the last sentence of the poem, he cautions “*It can be Important To have waited at least a moment to see what was already there*.” Given around 1 in 6 arthropod species carry *Wolbachia* at high prevalence, but male-killers are often at low frequency, PCR-based approaches may lead to these symbionts being missed; to borrow from Koch, one (s)train may hide another.

The core message of Koch’s poem is that overlooked things may actually be rather significant—and commonly more important than the thing first noticed. This is certainly the case for MK symbionts, as they are potent agents of evolutionary change. The act of killing male offspring results in a 2-fold cost: Killing c. 50% of the infected female’s offspring is combined with the Fisherian sex ratio selection produced to restore the rare sex. Together, these engender intense selection on the host to restore male viability. Indeed, one of the strongest cases of natural selection in an animal was the spread of a single genetic locus that suppressed MK activity by *Wolbachia* in the butterfly *Hypolimnas bolina*. In *H*. *bolina*, suppression increased to very high frequencies in under 5 years, such that the MK phenotype could no longer be detected in natural populations [7]. In the current paper, Richardson and colleagues present laboratory crossing and genomic data indicating the presence of dominant nuclear suppression of MK in *D*. *pseudotakahashii*. However, in contrast to *H*. *bolina*, suppression is rare in this system, as evidenced by the observation that the MK *Wolbachia* strain in natural populations was found *only* in females that gave rise to all-female broods (MK phenotype). If suppression were common, some suppressed MK *Wolbachia*-infected individuals would be expected to be found in the field sample. This then begs the question as to what forces are restricting its spread.

The rarity of suppression is interesting and useful. Low frequency of suppression is partly explainable by the low prevalence of infection—3.7% of females—but nevertheless, a suppressor would rescue 50% of the progeny of these females and represents—compared to other sources of selection—a very strong selection pressure with s ≈ 0.02 if suppression is cost free. Suppression spread can be inhibited if MK is rare, and a suppressed MK induces CI against the females that do not carry the strain [8]. While suppressed male flies carrying both infections (MK and CI) were incompatible with uninfected females, mating between males carrying the suppressed male-killer to females singly or coinfected produced a normal egg hatch rate. Thus, as females in the wild commonly carry at least one of the *Wolbachia* strains [4], these “rescued” males are compatible with the females they encounter naturally. The most likely hypothesis for suppression rarity is that suppression carries costs for flies not carrying the male-killer in the field context, preventing spread to high frequency. How this cost derives—in male or female or both sexes, and through the locus itself or through linked variants—is an important future avenue of research. Importantly, the combination of the polymorphic nature of male-killer and suppressor, and the lab tractability of the host species, will allow costs of suppression to be explored in this species.

The *D*. *pseudotakahashii* system provides an opportunity to investigate the mechanism of both MK and suppression. In the current paper, the genome of the male-killer is inferred from a doubly infected individual versus one carrying the CI strain alone; the data ruled out MK mediated through one candidate gene for MK—*Oscar* [9]—but was consistent with another—*wmk* [10]. To progress this work, the genome of the MK *Wolbachia* strain would need to be resolved from a singly infected individual followed by functional testing of candidates through transgenic expression. Signals of suppressor presence in the current paper are found through comparison of suppressed versus unsuppressed sublines, and the *Drosophila* system should afford the power to analyse these precisely in terms of mechanism. Altogether, the system provides a fascinating one for understanding both mechanism and field dynamics of MK/host interactions.

## References

[1] Vancaester E, Blaxter M. Phylogenomic analysis of *Wolbachia* genomes from the Darwin Tree of Life biodiversity genomics project. PLoS Biol. 2023;21(1):e3001972. doi: 10.1371/journal.pbio.3001972 36689552 PMC9894559

[2] Hurst GDD, Jiggins FM, Graf H, von der Schulenburg J, Bertrand D, West SA, et al. Male–killing *Wolbachia* in two species of insect. Proc R Soc Lond B. 1999;266(1420):735–740. doi: 10.1098/rspb.1999.0698

[3] Richardson KM, Ross PA, Cooper BS, Conner WR, Schmidt T, Hoffmann AA. A male-killing *Wolbachia* endosymbiont is concealed by another endosymbiont and a nuclear suppressor. PLoS Biol. 2023;21(4):e3001879. doi: 10.1371/journal.pbio.3001879 36947547 PMC10069767

[4] Richardson KM, Griffin PC, Lee SF, Ross PA, Endersby-Harshman NM, Schiffer M, et al. A *Wolbachia* infection from *Drosophila* that causes cytoplasmic incompatibility despite low prevalence and densities in males. Heredity. 2019;4:428–440. doi: 10.1038/s41437-018-0133-7 30139962 PMC6460763

[5] Jiggins FM, Bentley JK, Majerus ME, Hurst GD. How many species are infected with *Wolbachia*? Cryptic sex ratio distorters revealed to be common by intensive sampling. Proc Biol Sci. 2001;268(1472):1123–1126. doi: 10.1098/rspb.2001.1632 11375098 PMC1088716

[6] Koch K. One Train May Hide Another. From *One Train: Poems*, New York: Alfred A. Knopf, Inc.; 1994.

[7] Charlat S, Hornett EA, Fullard JH, Davies N, Roderick GK, Wedell N, et al. Extraordinary flux in sex ratio. Science. 2007;317(5835):214. doi: 10.1126/science.1143369 17626876

[8] Hornett EA, Engelstädter J, Hurst GD. Hidden cytoplasmic incompatibility alters the dynamics of male-killer/host interactions. J Evol Biol. 2010;23(3):479–487. doi: 10.1111/j.1420-9101.2009.01872.x 20040002

[9] Katsuma S, Hirota K, Matsuda-Imai N, Fukui T, Muro T, Nishino K, et al. A *Wolbachia* factor for male killing in lepidopteran insects. Nat Commun. 2022;13:6764. doi: 10.1038/s41467-022-34488-y 36376299 PMC9663696

[10] Perlmutter JI, Bordenstein SR, Unckless RL, LePage DP, Metcalf JA, Hill T, et al. The phage gene *wmk* is a candidate for male killing by a bacterial endosymbiont. PLoS Pathog. 2019;15(9):e1007936. doi: 10.1371/journal.ppat.1007936 31504075 PMC6736233

